# State-dependent diffusion of actin-depolymerizing factor/cofilin underlies the enlargement and shrinkage of dendritic spines

**DOI:** 10.1038/srep32897

**Published:** 2016-09-06

**Authors:** Jun Noguchi, Tatsuya Hayama, Satoshi Watanabe, Hasan Ucar, Sho Yagishita, Noriko Takahashi, Haruo Kasai

**Affiliations:** 1 Laboratory of Structural Physiology, Center for Disease Biology and Integrative Medicine, Faculty of Medicine, The University of Tokyo, Bunkyo-ku, Tokyo 113-0033, Japan; 2 CREST, Japan Science and Technology Agency, 4-1-8 Honcho, Kawaguchi, Saitama 332-0012, Japan

## Abstract

Dendritic spines are the postsynaptic sites of most excitatory synapses in the brain, and spine enlargement and shrinkage give rise to long-term potentiation and depression of synapses, respectively. Because spine structural plasticity is accompanied by remodeling of actin scaffolds, we hypothesized that the filamentous actin regulatory protein cofilin plays a crucial role in this process. Here we investigated the diffusional properties of cofilin, the actin-severing and depolymerizing actions of which are activated by dephosphorylation. Cofilin diffusion was measured using fluorescently labeled cofilin fusion proteins and two-photon imaging. We show that cofilins are highly diffusible along dendrites in the resting state. However, during spine enlargement, wild-type cofilin and a phosphomimetic cofilin mutant remain confined to the stimulated spine, whereas a nonphosphorylatable mutant does not. Moreover, inhibition of cofilin phosphorylation with a competitive peptide disables spine enlargement, suggesting that phosphorylated-cofilin accumulation is a key regulator of enlargement, which is localized to individual spines. Conversely, spine shrinkage spreads to neighboring spines, even though triggered by weaker stimuli than enlargement. Diffusion of exogenous cofilin injected into a pyramidal neuron soma causes spine shrinkage and reduced PSD95 in spines, suggesting that diffusion of dephosphorylated endogenous cofilin underlies the spreading of spine shrinkage and long-term depression.

Dendritic spines are postsynaptic to most excitatory synapses in the brain, and their structural changes are a major cellular basis for learning and memory. Spine generation and elimination are robust processes, even in the adult brain^1^,^2^,^3^, and spine enlargement and shrinkage play key roles in the plasticity of dendritic spines. We previously used two-photon glutamate uncaging to stimulate individual spines and demonstrated that dendritic spine enlargement underlay long-term potentiation (LTP) at the level of single spines^4^,^5^,^6^,^7^,^8^. We also reported that spine shrinkage and elimination can be induced when glutamate uncaging was paired with action potentials in the presence of GABAergic inhibition^9^.

Spine structures are dynamically maintained by the regulation of actin fibers (F-actins)^10^,^11^,^12^. Cofilin is a highly abundant actin-regulatory protein, which binds to and alters the physical properties of F-actin^13^,^14^. Dephosphorylated cofilin at its serine-3 residue (dp-cofilin) cleaves F-actin to generate new barbed ends that are sites for actin polymerization and depolymerizes F-actin at its pointed end to reduce fiber length. These bidirectional enzymatic activities of cofilin are inhibited by its phosphorylation at the serine-3 residue, as phosphorylated cofilin (p-cofilin) dissociates from F-actin^15^,^16^. Thus, the entire lifecycle of F-actin is regulated by cofilin. Dendritic spine enlargement and shrinkage are likely also regulated by the phosphorylation^9^,^12^ and dephosphorylation^9^,^17^ of cofilin^18^. We previously showed that bidirectional structural plasticity is controllable in individual spines by modulating cofilin phosphorylation^9^.

It has been reported that spine shrinkage tends to spread^9^,^19^ as in the case with LTD^20^,^21^,^22^. In contrast, spine enlargement is confined to the stimulated spine^4^,^5^,^6^,^7^,^8^ as with LTP^21^,^23^. If both processes are regulated by cofilin, the diffusional properties of cofilin, which can be assessed by its retention time in the spine, must differ during enlargement and shrinkage. Recently, cofilin has been shown to accumulate during spine enlargement, and long-term spine enlargement was inhibited by shRNA-mediated cofilin knockdown^12^. Both constitutively active and inactive-phosphomimetic cofilin mutants failed to resume long-term spine enlargement and the cofilin accumulation^12^. Considering phosphorylated cofilin cannot bind with F-actin directly, the accumulated cofilin was thought to be dephosphorylated before binding F-actin, although both phosphorylation and re-dephosphorylation of cofilin are necessary for long-term spine enlargement^12^. On the other hand, an accumulation of phosphorylated cofilin was reported after LTP stimulation using immunohistochemistry^24^. To resolve these discrepant findings, the objective of this study was to address whether dephosphorylated or phosphorylated cofilin accumulates in the stimulated spine after enlargement. In addition, we sought to determine whether spine shrinkage is generated only by the diffusion of dephosphorylated cofilin along the dendrite.

We investigated cofilin diffusion using photoactivatable green fluorescent protein (PAGFP) to probe the various cofilin-1 states. We then determined whether dp-cofilin, which was infused into the soma of pyramidal neurons, could spread and induce spine shrinkage and whether this spine shrinkage was associated with a reduction in PSD95 levels. The data suggest that spine shrinkage is proportionally associated with a reduction in postsynaptic density (PSD), unlike spine enlargement^12^. Thus, we demonstrate that cofilin activity and diffusion differs between enlargement and shrinkage and that cofilin is the major spatial organizer of the structural plasticity of dendritic spines.

## Results

### Cofilin diffusion along dendrites and spines in the resting state

To investigate whether the cofilin serine-3 phosphorylation state affects its diffusion properties, we first assessed diffusion at the resting state. A simple hypothesis is that spine enlargement is due to the lack of diffusion of p-cofilin along dendrites, which prevents depolymerization of F-actins at individual spines. The prevention of F-actin depolymerization may enhance elongation of the actin filaments and the spine enlargement. To test this hypothesis, we investigated the manner in which cofilin diffusion was affected by its phosphorylation. We determined the spread of cofilin proteins along dendrites in hippocampal slice cultures using PAGFP-cofilin fusion proteins^10^,^25^. Cofilin protein is activated by dephosphorylation on its serine-3 residue. To investigate the properties of dp- and p-cofilin separately, we used a serine-3 to alanine (S3A) and a serine-3 to glutamate (S3E) cofilin mutant, which are constitutively active and inactive-phosphomimetics, respectively^13^,^26^.

**Figure 1:**
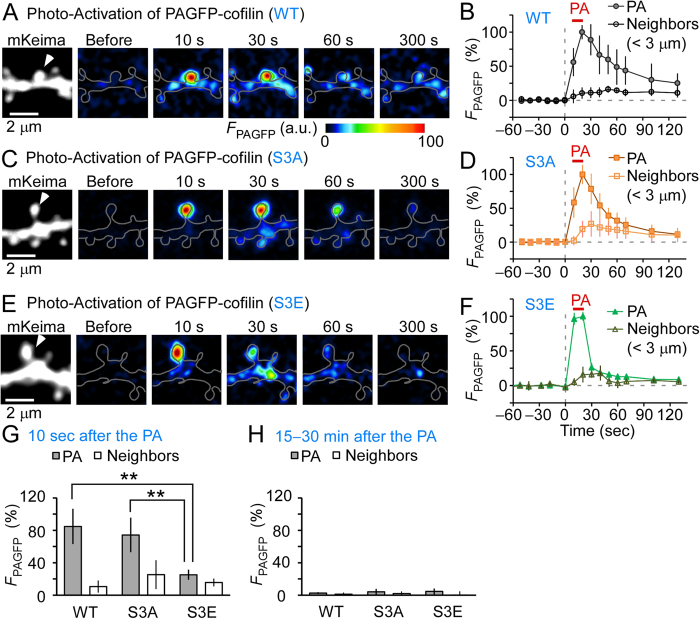
Spreading of cofilin along dendrites in the resting state. Photoactivatable green fluorescent protein (PAGFP)-cofilin fusion proteins were photoactivated (ten times at 5 Hz for 1 ms each, repeated twice with a 10 s interval). (**A,C,E**) Images of dendrites expressing mKeima and wild-type (WT) PAGFP-cofilin (**A**), PAGFP-(S3A) cofilin (**C**), or PAGFP-(S3E) cofilin (**E**). White arrowheads mark an individual photoactivated spine. (**B,D,F**) Time course of PAGFP fluorescence in photoactivated (PA) and neighboring spines (*n* = 6, 6, and 7 dendrites for WT, S3A, and S3E, respectively). (**G,H**) Fluorescence intensities of PAGFP-cofilin at 10 s (**G**) or 15–30 min (**H**) after photoactivation. Ten seconds after terminating PA, the fluorescence intensity was lower in dendrites expressing S3E (25% ± 6.1%) than in WT- (85% ± 22%, *p* < 0.01) and S3A-expressing dendrites- (74% ± 21%, *p* < 0.01). In contrast, at 15–30 min, no differences were observed (2.6% ± 1.1%, 4.3% ± 3.4%, and 4.6% ± 3.5% for WT, S3A, and S3E, respectively; *p* > 0.4). Data represent the mean ± SD. **p < 0.01, using Steel’s multiple comparison test after the Kruskal–Wallis test.

In the resting state, following PA, we found that wild-type (WT) cofilin diffused from the spine with a time constant of 41 ± 13 s, spread to neighboring spines, and was then diluted along dendrites (Fig. 1A,B). Similar findings were obtained for S3A cofilin (21 ± 2.3 s; Fig. 1C,D). In contrast, S3E cofilin more rapidly diffused (5.4 ± 0.4 s; Fig. 1E,F; *p* < 0.0075). Thus, PAGFP fluorescence (*F*_PAGFP_) at 10 s after PA in the irradiated spine was significantly smaller for S3E mutants than for WT or S3A mutants (Fig. 1G). The difference was negligible for residual *F*_PAGFP_ after a sufficient dilution period (15–30 min after PA) (Fig. 1H). These findings suggest that the diffusion of WT or S3A cofilin, but not S3E cofilin, was significantly impeded by their binding to F-actins^13^,^26^. However, as all cofilin forms diffused quickly along dendrites in this experiment, these data indicate that retention of p-cofilin in individual spines in the resting state fail to account for the previously observed confined spine enlargement.

### Confinement of cofilins within a spine during spine enlargement

**Figure 2:**
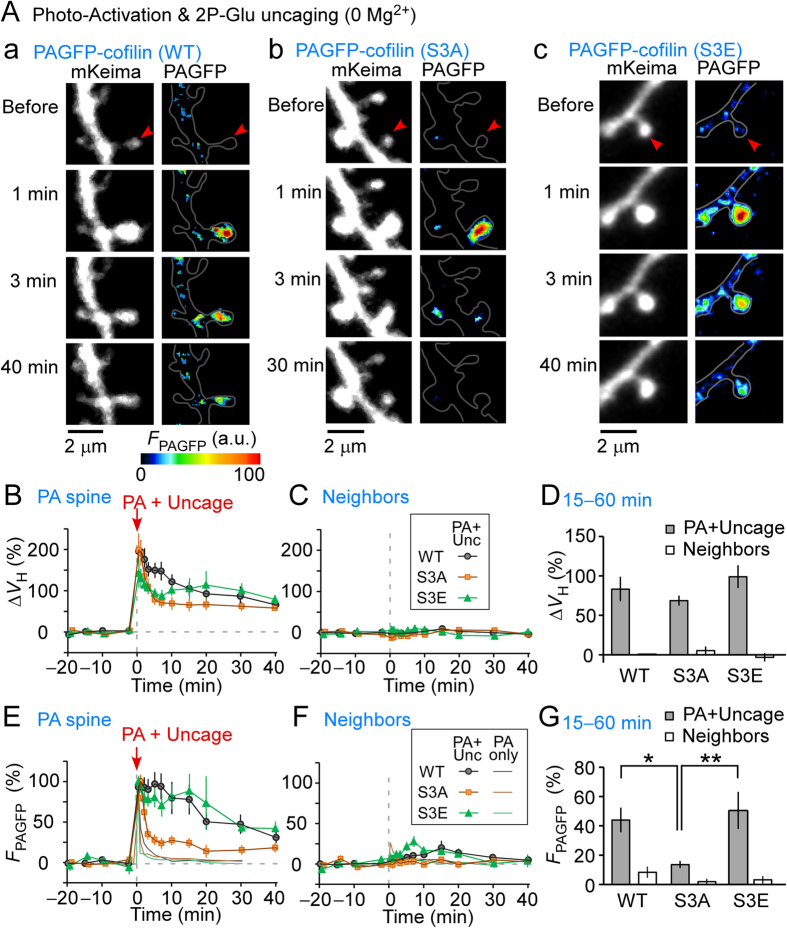
Spreading of cofilin along dendrites during spine enlargement. Spine enlargement was induced by repetitive glutamate uncaging (0.6 ms, 5 Hz, 80 times), in which incidental light activated the photoactivatable green fluorescent protein (PAGFP)-cofilin fusion proteins. (**A**) Images of dendrites expressing mKeima and PAGFP-cofilin (A-a), PAGFP-(S3A) cofilin (A-b), and PAGFP-(S3E) cofilin (A-c). Red arrowheads mark an individual stimulated spine. (**B,C**) Time course of spine enlargement in stimulated, photoactivated (PA) (**B**) and neighboring (**C**) spines expressing either wild-type (WT) PAGFP-cofilin (nine dendrites), PAGFP-(S3A) cofilin (ten dendrites), or PAGFP-(S3E) cofilin (six dendrites). (**D**) Amplitudes of spine enlargement in PA and neighboring spines at 15–60 min after uncaging. There were spine volume changes of 83% ± 15%, 69% ± 6.2%, and 99% ± 14% of WT (nine dendrites), S3A (ten dendrites), and S3E (six dendrites), respectively, (stimulated spine, *p* > 0.1). (**E,F**) Time course of PAGFP fluorescence in stimulated (**E**) and neighboring (**F**) spines, expressing WT PAGFP-cofilin, PAGFP-(S3A) cofilin, or PAGFP-(S3E) cofilin. The results of ‘no uncaging’ controls were superimposed (thin lines) when PA was performed ten times without caged glutamate for 1 ms at 5 Hz, repeated twice with a 10 s interval (Fig. 1). (**G**) Fluorescence intensities of PAGFP in PA and neighboring spines at 15–60 min after uncaging. A difference in residual intensities of *F*_PAGFP_ between WT, S3A, and S3E of stimulated spines at 15–60 min after uncaging was evident (44% ± 8.4%, and 14% ± 2.4%, and 51% ± 13% for WT, S3A, and S3E, respectively) (*p* < 0.05 vs. WT and p < 0.01 vs. S3E). In contrast, no difference was observed between neighbors that showed little enlargement. Error bars represent the mean ± SEM.

We examined the diffusive properties of WT, S3E, and S3A cofilin mutants during spine enlargement. We used low cofilin expression levels so as not to affect spine enlargement, which was induced by repetitive glutamate uncaging in Mg^2+^-free solution (Fig. 2A–D). As shown in Fig. 2D, the spine volume increment was similar in the stimulated spines expressing all mutants. WT and the S3E PAGFP-cofilin mutant accumulated in the stimulated spines for 30 min after spine enlargement (Fig. 2A,E,G), similar to previous observations of the stable enlargement of the F-actin pool^10^. Such an accumulation was not detected in neighboring spines that were not enlarged (Fig. 2C,F). In contrast, S3A PAGFP-cofilin more rapidly diffused from both the enlarged spine (Fig. 2A,E,G) and resting spines (Fig. 2E, orange thin line). The slightly delayed S3A cofilin diffusion (Fig. 2E, orange square, time constant, 59 ± 8.4 s) compared to the ‘no uncage’ control (thin orange line, 21 ± 2.3 s, *p* = 0.0024, Mann–Whitney rank sum test) suggests that S3A cofilin was trapped in enlarged spines to some degree, likely by F-actin enrichment. These data indicate that the LTP protocol induced a selective accumulation of p-cofilin and imply that spine enlargement generated a molecular scaffold that trapped p-cofilin within spines.

### Effect of phosphorylated cofilin on spine enlargement

**Figure 3:**
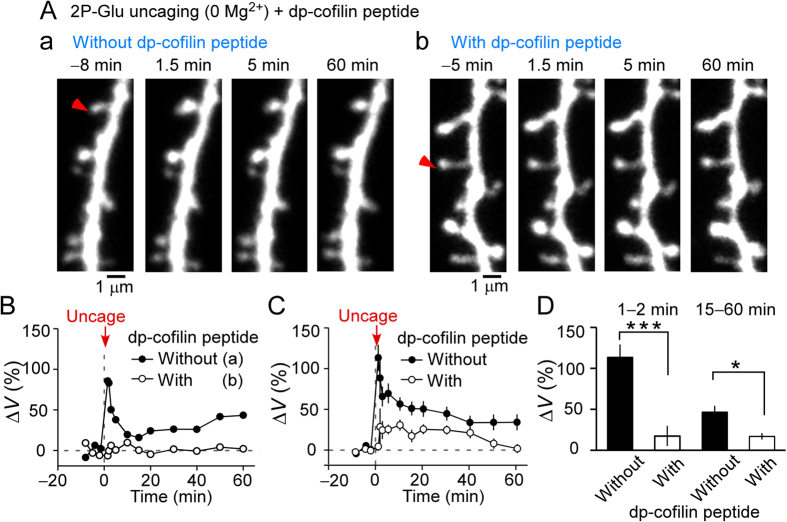
Effects of a dephosphorylated cofilin (dp-cofilin) peptide on spine enlargement. (**A**) Images of a dendrite stimulated by repetitive uncaging of caged glutamate. Pyramidal neurons were whole-cell patch clamped and perfused without (A-a) or with (A-b) dp-cofilin peptide. Red arrowheads mark stimulated dendritic spines. (**B**) Time course of the changes in spine volumes from (A-a) and (A-b). (**C,D**) Time course of the changes in spine volumes and averaged amplitudes of spine enlargement during 1–2 min (without the dp-cofilin peptide, 113% ± 15%, 27 spines; with peptide, 17% ± 12%, 19 spines) or 15–60 min (without peptide, 46% ± 7.7%, 31 spines; with peptide, 17% ± 3.7%, 19 spines) after inducing spine enlargement. Data represent the mean ± SEM. ***p < 0.0001, *p = 0.016 (Mann–Whitney rank sum test).

We investigated whether cofilin phosphorylation was required for spine enlargement, which was induced by repetitive glutamate uncaging in external Mg^2+^-free solution (Fig. 3A–D). For this purpose, we used a dp-cofilin peptide, which blocks endogenous cofilin phosphorylation^17^. Spine enlargement was greatly reduced in the early phase (1–2 min after stimulation), and enlargement was still reduced during the late phase (15–60 min) (Fig. 3D). The early phase appeared more potently blocked than the late phase, which is the opposite of the effect observed with an inhibitor or mutation of CaMKII^4^,^7^. This suggests that the early phase is more dependent on the blockade of F-actin depolymerization by cofilin rather than CaMKII activation.

Our data are consistent with previous results showing the blockade of enlargement under spike-timing dependent plasticity conditions^9^ and in cells expressing GFP-cofilin^12^. Enlargement was not completely abolished by the dp-cofilin peptide, and this likely is due to either an incomplete effect of the peptide or a mechanism that induced spine enlargement independent of cofilin phosphorylation. Our results suggest that cofilin phosphorylation is the major regulator of spine enlargement, even following the most potent induction protocol for spine enlargement.

### Spread of spine shrinkage and cofilin diffusion

Unlike spine enlargement, spine shrinkage, induced by the spike-timing dependent protocol, spreads along dendrites and is blocked by a p-cofilin peptide, indicating that it is caused by cofilin dephosphorylation^9^. To investigate whether spine shrinkage spread in intact cells not subjected to whole-cell perfusion, we induced spine shrinkage using low-frequency repetitive stimulation with glutamate uncaging (LFS; 1 Hz, 300 times) of cells that were labeled with PSD95-mGFP and mKeima. We found that effective spine shrinkage could be induced by LFS and was accompanied by a parallel reduction in PSD95 fluorescence (Supplementary Fig. S1). This is in contrast to spine enlargement, which was not associated with increased PSD95 levels, particularly during early time points following stimulation^12^. Importantly, like the reduction in PSD95 levels, spine shrinkage spread to the neighboring spine (Supplementary Fig. S1). At 60–80 min after stimulation, spine shrinkage of the stimulated spine (*p* < 0.018, Wilcoxon signed-rank test against zero) spread to neighboring spines within 3 μm, which was true for spine shrinkage that was induced by the spike-timing dependent protocol. However, for neighbors further than 3 μm from the stimulated spine, the volume reduction was not significant (Supplementary Fig. S1).

**Figure 4:**
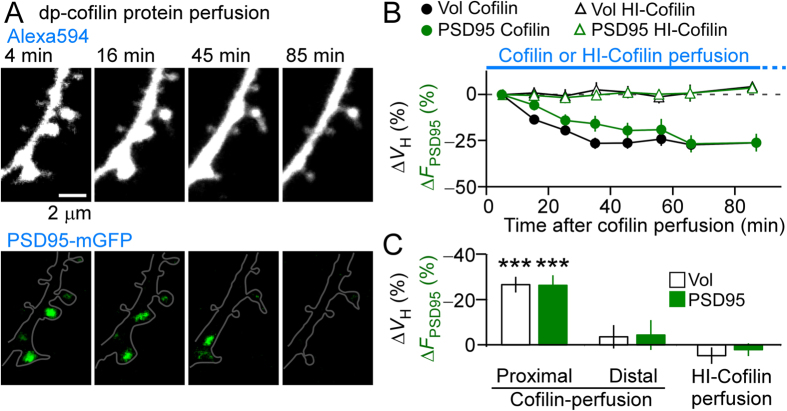
Effects of whole-cell perfusion of dp-cofilin on dendritic spines. (**A**) Fluorescence images of Alexa Flour 594- and PSD95-mGFP-labelled spines, which were induced to shrink using whole-cell perfusion of 10 μM recombinant human cofilin-1 protein. (**B**) Time course of the changes in spine volumes and PSD95-mGFP fluorescence intensities in cells injected with cofilin (76 spines, five cells) or with heat-inactivated (HI)-cofilin (78 spines, three cells). (**C**) Average reductions in spine volumes and PSD95-mGFP fluorescence intensities of cells perfused with cofilin at the proximal dendrite (40–130 μm from the soma, five cells, 76 spines), at the distal dendrite (230–430 μm, 38 spines), or perfused with HI-cofilin (at the proximal dendrite, 78 spines, three cells). The reductions in spine volumes and the fluorescence intensity of PSD95-mGFP were significant in the proximal dendrites (−27% ± 3.5%, ***p < 0.0001, Wilcoxon signed-rank test vs. zero and −26% ± 4.4%, ***p < 0.0001) but not in the distal dendrites [−3.5% ± 5.2% (*p* = 0.18) and −4.4% ± 6.6% (p = 0.24)) or HI-cofilin perfusion [4.8% ± 3.6% (*p* = 0.52) and 2.1% ± 2.8% (*p* = 0.68)]. Data represent the mean ± SEM.

We could not conduct a PAGFP-cofilin spread analysis (e.g. Figs 1 and 2) to examine directly the effect of cofilins during spine shrinkage since fluorescence background is high after the long-term repetitive stimulation. Instead, we took advantage of whole-cell perfusion to introduce a dp-cofilin protein (full-length recombinant, 10 μM) into the soma of pyramidal neurons and investigated spines in the ternary dendrites, which were used in spine enlargement experiments. Within 30 min, numerous shrunken spines with reduced PSD95 levels were observed along the dendritic tree at <150 μm from the soma (Fig. 4A,B). Moreover, the reduction in spine volume at 60 min after introducing the patch-clamp was much larger in the proximal spines, which were located 40–130 μm from the soma compared with the distal spines (230–430 μm). Moreover, heat-inactivated cofilin (HI-cofilin) did not detectably induce these effects (Fig. 4C). Similarly, following dp-cofilin introduction, the reduction of PSD95-mGFP fluorescence was larger in the proximal spines compared to the reduction in distal spines or to the reduction caused by HI-cofilin (Fig. 4C).

These experiments indicate that exogenous cofilins diffused along a dendrite and induced the spreading of spine shrinkage, similar to that induced by LFS (Supplementary Fig. S1) or spike-timing dependent induction^9^. Because dp-cofilin is the most abundant cofilin form in the cytosol^16^, an interesting question is why dp-cofilin perfusion was so effective in shrinking spines. A reasonable explanation is that the amounts of F-actin, G-actin, p-cofilin, dp-cofilin, and other G-actin-binding proteins are locally balanced, and a slight excess of dp-cofilin reduces F-actin content^16^. Thus, it is likely that the spreading of spine shrinkage is mediated by the cofilin diffusion. Moreover, cofilin injections sometimes translocated PSD95 clusters (Fig. 4A, lower panels), suggesting that PSD anchoring to spines requires F-actins.

## Discussion

We investigated cofilin diffusion along dendrites and found that it was state-dependent and tightly confined to the stimulated spine only when p-cofilin was induced by spine enlargement. Our data support a role of p- and dp-cofilin in spine enlargement and shrinkage, respectively. In addition, we determined that shrinkage spread, even in cells that were not subjected to whole-cell clamping. The same relationships hold for LTP and LTD^21^,^22^,^23^. Spine enlargement and LTP require stronger stimulation compared with those for spine shrinkage and LTD. Collectively, our data suggest a key role for cofilin in mediating dendritic spine plasticity, which would be expected to impact downstream LTP and LTD processes, and thus, learning and memory.

Cofilin function in spine enlargement has been previously reported^12^. Like our results, the previous study showed that dp-cofilin peptide prevented spine enlargement after repetitive glutamate uncaging. However, the previous study also showed a contradictory elevation of cofilin activity after the induction of spine enlargement using FRET^12^. Furthermore, cofilin protein knockdown using shRNA followed by exogenous S3A or S3D mutant expression failed to rescue the inhibition of the structural LTP. Thus, the author speculated that a phosphorylation/dephosphorylation cycle is required for proper spine enlargement. However, we showed that an inactive phosphomimetic mutant (S3E) can be retained in the stimulated spine during enlargement, perhaps because in our study, endogenous cofilin/ADF is intact. In addition, the previous paper showed that the constitutively active cofilin mutant (S3A) accumulated in spines shortly after LTP induction, but this localization was lost quickly, which is consistent with our study. Thus, the current and previous studies both support the notion that p-cofilins do not cleave or depolymerize F-actins, resulting in elongated F-actins that enlarged spines. Moreover, the accumulation of p-cofilin-containing molecular aggregates at the spine base may serve as a scaffold for F-actin elongation (see below).

Our data suggest that LTP stimulation phosphorylates cofilin and that p-cofilin accumulates in stimulated spines. Moreover, p-cofilin was confined to the spine only when the LTP induction protocol was applied. One possible explanation of these findings is that p-cofilin may form a complex with F-actin in conjunction with other proteins, as a direct binding between p-cofilin and actin has not been reported. Active CaMKII, RhoA and Rac1 generate several phosphorylated proteins, including LIMK, PAK, and slingshot^27^,^28^,^29^,^30^, whereas spine enlargement involves a recruitment of proteins, including CaMKII^4^,^7^, RhoA, cdc42^31^, and myosinII^32^,^33^. Furthermore, LTP induction accumulates phosphorylated proteins such as p-cofilin, p-PAK, p-FAK, and integrin^24^,^30^,^34^, which are involved in assembling stress fibers^26^,^35^. Collectively, these studies suggest that the spine-anchored stable F-actin may represent a high-order molecular complex–similar to stress fibers that are required for input-specific modification of synapses–which can bind p-cofilin.

We suggest that LTP stimulation of a single spine does not dephosphorylate cofilin, despite the stronger stimuli required to mediate LTP compared to stimuli required for LTD, as shrinkage in neighboring spines is undetectable^4^,^5^,^6^,^7^,^8^. Thus, it is likely that cofilin phosphorylation and dephosphorylation are competitively induced, and once phosphorylation is induced by larger increases in cytosolic Ca^2+^ concentrations, cofilin is not dephosphorylated. This spine-specific regulation may not hold when many spines are simultaneously stimulated to induce LTP and the synapses which neighbor stimulated spines show shrinkage^36^ and LTD^37^.

Despite its binding to F-actins, we demonstrated that dp-cofilin efficiently diffused along dendrites, enabling heterosynaptic shrinkage of spines along dendrites, which was consistent with the spread of LTD along dendrites^20^,^21^,^22^. Moreover, we demonstrated that PSD95 clusters shrunk and were occasionally displaced by exogenous cofilin. Cofilin depolymerizes and cleaves F-actins^15^. Thus, cofilin may impair and destabilize the critical F-actin that is polymerized at PSDs. The proportional reduction in PSD size during spine shrinkage is consistent with the simultaneous reduction of the AMPA and NMDA components of excitatory postsynaptic potentials (EPSPs) after LTD induction^38^,^39^. In contrast, changes in PSD are not proportionally induced during spine enlargement but occur after a delay of approximately 30 min^12^. Consistently, the AMPA, but not NMDA, component of EPSPs is affected by LTP^40^,^41^,^42^.

Our data suggest that cofilin enlarges and shrinks spines, which is a very efficient mechanism for creating bidirectional structural plasticity. We propose that, firstly, p-cofilin (or dp-cofilin) generation naturally reduces dp-cofillin (or p-cofilin) and selectively induces either stimulated spine enlargement or shrinkage. Secondly, p-cofilin is confined to enlarged spines to support the altered geometry of individual spines via a similar structure to stress fibers. Thirdly, synapses can communicate with each other by dp-cofilin diffusion and competition with p-cofilin, so that only spines that are more efficiently stimulated relative to neighboring spines can survive. In this manner, the effects of weak stimulation (LFS or the LTD protocol) induced spine shrinkage spread among spines. This is in contrast to the effects of stronger stimulation (high frequency stimulation or LTP protocol), which were highly confined to stimulated spines. Thus, the unexpected cofilin diffusional properties mediate an asymmetric organization of spine enlargement and shrinkage and play a key role in the efficient competitive selection of spines along dendrites. Although speculative, our data suggests a role for phosphorylated cofilin in retaining some synapse-specific information for short periods of time (e.g. ≤1 hour) during the establishment of memory and learning, which can be reversed by its dephosphorylation.

## Methods

### Preparation of slice cultures

All animal procedures were approved by the Animal Experiment Committee of the University of Tokyo. Procedures were in accordance with the University of Tokyo’s Animal Care and Use Guidelines. Hippocampal slices (350 μm thick) were prepared from 6- to 8-day-old Sprague Dawley rats (males and females), mounted onto 0.4 μm culture-plate inserts (EMD Millipore), and incubated at 35 °C and 5% CO_2_ in a medium comprising 50% minimum essential media, 25% Hanks’ balanced salt solution, 25% horse serum (Gibco), and glucose (6.5 g/L). After 6–8 days in culture, the slices were transfected with a Gene Gun system (PDS-1000; Bio-Rad, Hercules, CA). Imaging experiments were performed 2–7 days after transfection. Slices were individually transferred to recording chambers and superfused with an artificial cerebral spinal fluid (ACSF) containing 125 mM NaCl, 2.5 mM KCl, 2 mM CaCl_2_, 1 mM MgCl_2_, 1.25 mM NaH_2_PO_4_, 26 mM NaHCO_3_, and 20 mM glucose, which was bubbled with 95% O_2_ and 5% CO_2_. Bathing solutions contained 200 μM Trolox (Sigma-Aldrich). For the spine enlargement experiments (Figs 2 and 3), the bathing solution contained 125 mM NaCl, 2.5 mM KCl, 3 mM CaCl2, zero mM MgCl2, 1.25 mM NaH2PO4, 26 mM NaHCO3, 20 mM glucose, and 1 μM tetrodotoxin. Hippocampal CA3 regions were removed to reduce burst firing. All physiological experiments were performed at 30 °C–32 °C.

### Two-photon excitation imaging and photoactivation

Two-photon imaging of dendritic spines was performed using an upright microscope (BX61WI; Olympus) equipped with an FV1000 laser scanning microscope system (FV1000, Olympus) and a water-immersion objective lens (LUMPlanFI/IR, 60×, NA 0.9)^43^. The system included two mode-locked, femtosecond-pulse Ti:sapphire lasers (MaiTai from Spectra Physics), one set at a wavelength of 720 nm and the other at 830 nm for Alexa Fluor 594 or 910 nm for mKeima, mGFP, and PAGFP. Each laser was connected to the microscope via an independent scan head and gated using an acousto-optic modulator for two-photon uncaging of caged glutamate and imaging.

The second or third dendritic branch was used for imaging and uncaging experiments. Three-dimensional reconstructions of dendritic morphology were generated by the summation of fluorescent values at each pixel in 17–29 *xy* images, with each separated by 0.5 μm. After whole-cell perfusion, the fluorescence intensities of dendrites gradually increased for 20 min and were corrected according to the entire fluorescence of a dendritic region. The volumes of spine heads were estimated from the total fluorescence intensity. ‘Neighboring’ spines were spines within 3 μm from stimulated spines, unless otherwise stated. Spines that changed volume >30% before uncaging were excluded from the data analysis^4^.

Two-photon activation of PAGFP-cofilin was performed using slice preparations that were transfected with PAGFP-cofilin and mKeima. PA of PAGFP-cofilin at 720 nm was induced ten times at a single point in a spine for 1 ms at 5-Hz pulses, which was repeated twice with a 10 s interval. Three-dimensional images of dendrites were acquired at 10 s intervals. The power of the PA laser was set from 6 to 10 mW. In the enlargement experiments, PA of PAGFP-cofilin was simultaneously induced 80 times with the uncaging of CDNI-glutamate at 720 nm for 0.6 ms at 5-Hz pulses. The fluorescence of photoactivated PAGFP-cofilin and mKeima was excited at 910 nm, and emitted fluorescence was acquired at 500–560 nm and 590–680 nm, respectively. There was a low level of bleed-through of mKeima to the PAGFP channel (1.7%) and of PAGFP to the mKeima channel (0.6%). These values were subtracted from quantitative analyses.

### Electrophysiology and glutamate uncaging

For normal whole-cell recordings, patch-clamp electrodes (open tip resistance, 4–7 MΩ) were filled with 138 mM potassium gluconate, 4 mM MgCl_2_, 10 mM disodium phosphocreatine, 50 μM Alexa 594 (Life Technologies Corporation), 4 mM ATP (sodium salt), 0.4 mM GTP (sodium salt), 10 mM Hepes-KOH (pH 7.2), 0.5 mM K-EGTA, and 5 μM β-actin (human platelet; Cytoskeleton). Dp-cofilin peptide (MASGVAVSDGVIKVFN, 0.5 mM, BEX) was dissolved in pipette solutions. Cofilin protein (bacterially expressed, human full-length recombinant cofilin-1, consisted mainly of dephosphorylated cofilin (dp-cofilin), Cytoskeleton) was dissolved at 10 μM to perfuse the neurons using whole-cell patch clamping. Cofilin (HI-cofilin) was inactivated by boiling at 95 °C for 15 min. Series resistance was 21.5 ± 4.1 MΩ (mean ± SD), and the resting membrane potential was −59.5 ± 3.4 mV (mean ± SD). Cells were voltage clamped at −65 mV (Axopatch 200B, Molecular Devices). Cells with resting potentials at more than −53 mV at uncaging were excluded from data analyses. Currents were evoked 3–5 times at each time, low-pass filtered at 2 kHz, sampled at 10 kHz, and averaged.

CDNI-glutamate (2 mM, Nard Institute Ltd.)^44^,^45^ was locally puffed from glass pipettes near selected dendrites. Selective photolysis of CDNI-glutamate was performed using femtosecond lasers at 720 nm (0.6 ms, unless otherwise stated). Photo-released glutamate levels were adjusted by changing the laser powers (approximately 6 mW). In the experiments designed to measure spine shrinkage, we added 200 nM muscimol to the puffing solution to stabilize the membrane potential of neurons that were not subjected to whole-cell clamping.

### Plasmid construction

The human cytomegalovirus immediate-early promoter of the pEGFP-N1 vector (Clontech) was replaced between the NdeI and NheI sites with a CAG promoter generated by polymerase chain reaction (PCR) (forward primer 5′-aatgacgtatgttcccatagtaacgcc-3′; reverse primer 5′-ctagctagctctttgccaaaatgatgagacagcaca-3′) to generate pCAG-EGFP-N1. The EGFP region of pCAG-EGFP-N1 was replaced between the NheI and BsrGI sites with PAGFP cDNA^10^,^25^, followed by the insertion of another PAGFP cDNA between the BamHI and Age I sites to obtain the pCAG-tandem PAGFP-N1 vector. The monomeric version of a GFP vector^46^, pCAG-mGFP-N1, was generated from pCAG-EGFP-N1 using PCR mutagenesis with a PrimeSTARMax DNA polymerase mutagenesis protocol (Takara Bio Inc.; forward primer 5′-cagtccaagctgagcaaagaccccaacgagaag-3′; reverse primer 5′-gctcagcttggactgggtgctcaggtagtggttg-3′). A cDNA library was prepared from rat prefrontal cortex using commercial kits (RNeasy Protect Mini kit, QIAGEN GmbH; SuperScript First-Strand Synthesis System, Life Technologies). Rat cofilin-1 and rat PSD-95 cDNAs were amplified by PCR from the cDNA library using PrimeSTAR Max DNA polymerase (Takara Bio Inc.; forward primer for rat cofilin-1:5′-cctttcgaat tccggaaacatggcctctggtg-3′; reverse primer 5′-gtggatccaaaggcttgccctccagggaaatgac-3′; forward primer for rat PSD95: 5′-ccaagcttgccaccatggactgtctctgtatagtgac-3′; reverse primer 5′-ggaccggtccgagtctctctcgggctgggacccagat-3′). Amplified cDNAs were ligated to pCAG-tandem PAGFP-N1 and pCAG-mGFP-N1, respectively, to yield the expression vectors pCAG-rat cofilin-1 tandem PAGFP (EcoRI-BamHI) and pCAG-rat PSD-95-mGFP (Hind III-Age I). The rat S3A and S3E cofilin mutants were generated using PCR mutagenesis as stated above (forward primer for S3A: 5′-atggccgccggtgtggctgtctctgatggagtc-3′; reverse primer 5′-acaccggcggccatgtttccggaattcgaagc-3′; forward primer for S3E: 5′-atggccgagggtgtggctgtctctgatggagtc-3′; reverse primer 5′-acaccctcggccatgtttccggaattcgaagc-3′). To construct the mKeima vector (pCAG-mKeima), the EGFP region of pCAG-EGFP-C1 was replaced between the NheI and Mfe I sites with hmKeima-Red cDNA (MBL Inc.).

### Statistical analysis

All data are presented as mean ± SEM. (*n* = spine numbers), unless otherwise stated. Statistical tests were performed among spines or dendrites, as indicated. In Figs 1G,H and 2D,G, data were first analyzed using the Kruskal–Wallis test followed by Steel tests for multiple comparisons. The Mann–Whitney rank sum test was used to analyze the data shown in Fig. 3D, and the Wilcoxon signed-rank test was used for data in Fig. 4C. Other statistical tests are identified in the text. Data collection and analysis were not performed in a blinded manner, and data were not randomized for analysis. No statistical methods were used to predetermine sample sizes, although our sample sizes are similar to those previously reported^4^,^5^,^6^,^7^,^8^.

## Additional Information

**How to cite this article**: Noguchi, J. *et al*. State-dependent diffusion of actin-depolymerizing factor/cofilin underlies the enlargement and shrinkage of dendritic spines. *Sci. Rep.* **6**, 32897; doi: 10.1038/srep32897 (2016).

## Supplementary Material

Supplementary Information
