# Supplementary material for: State-dependent diffusion of actin-depolymerizing factor/cofilin underlies the enlargement and shrinkage of dendritic spines
Source: Sci Rep. 2016 Sep 6;6:32897. doi: 10.1038/srep32897 (PMC5011767; doi:10.1038/srep32897)
Supplement: Supplementary Information [file srep32897-s1.pdf]

State-dependent diffusion of actin-depolymerizing factor/cofilin underlies the enlargement and shrinkage of dendritic spines

Jun Noguchi, Tatsuya Hayama, Satoshi Watanabe, Hasan Ucar, Sho Yagishita, Noriko Takahashi & Haruo Kasai

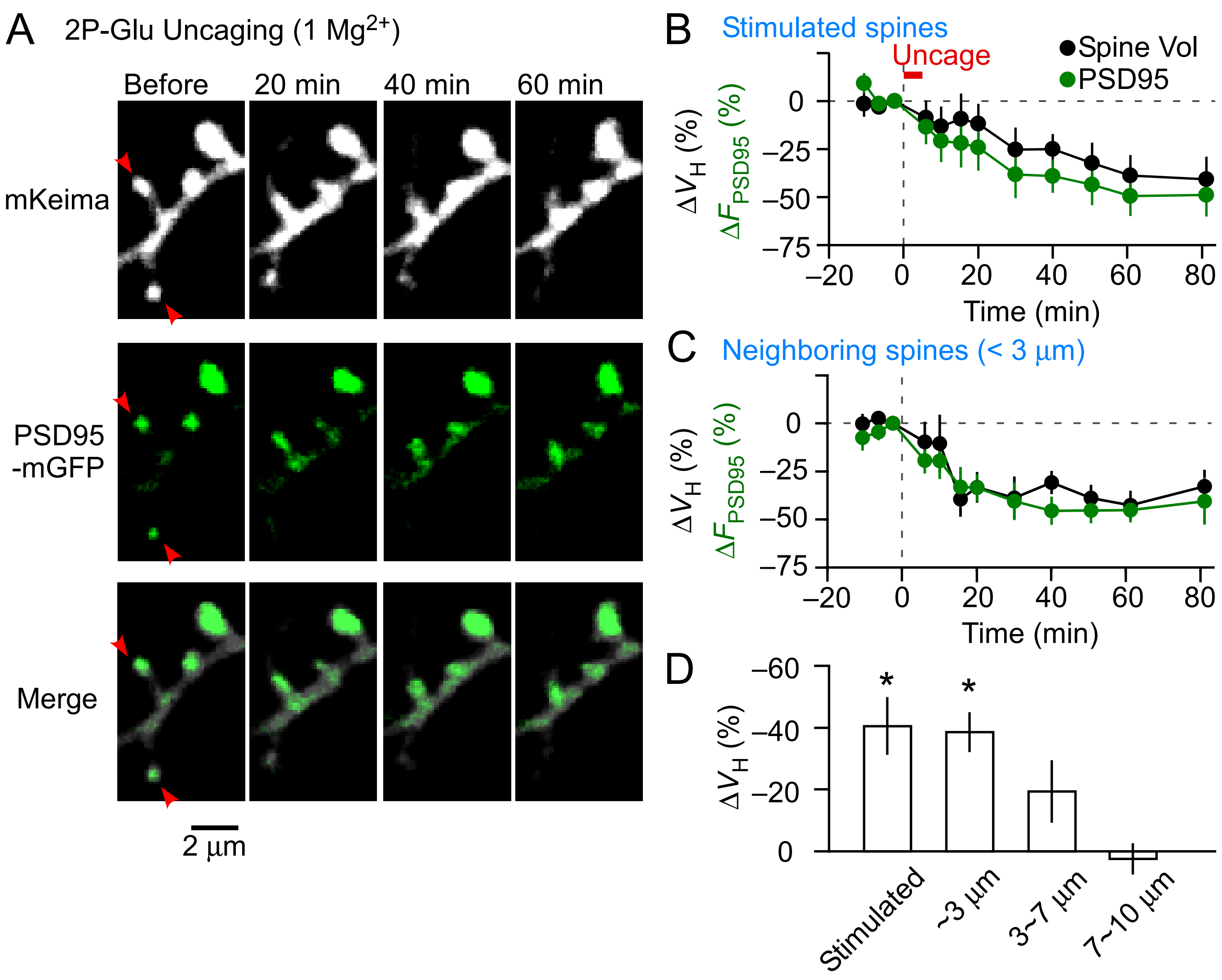

Supplementary Figure S1. Spine shrinkage induced by low-frequency uncaging stimulation (LFS).

Glutamate uncaging was applied at 1 Hz for 5 min in a slice preparation that was transfected with mKeima and PSD95-mGFP. LFS via glutamate uncaging was applied to non-whole-cell clamped neurons, and spine volume changes were measured according to mKeima fluorescence. (**A**) Images of a dendrite where two spines, which are indicated by the red arrows, were subjected to LFS. The upper and lower panels show mKeima and PSD95-mGFP, respectively. (**B, C**) Time course of the change in spine volumes and PSD95-mGFP fluorescence of spines subjected to LFS (red bar, eight spines, five dendrites) (B) or those in the neighboring spines (< 3 µm from the stimulated spine, seven spines) (C). (**D**) The average reduction in spine volumes at each distance from the stimulated spines (stimulated, –41% ± 9.4%, eight spines, five dendrites; < 3 µm, 39% ± 6.4%, seven spines; 3–7 µm, –19% ± 10%, 15 spines; 7–10 µm, 2.5% ± 5.1%, eight spines). The volume reduction was significant according to the Wilcoxon signed-rank test vs. zero for < 3 µm (p = 0.018) but was not significant for 3–7 µm and 7–10 µm at p = 0.088 and 0.58, respectively. Data represent the mean ± SEM, *$p < 0.05$.
